# Muscular and hepatosplenic candidiasis in a patient with acute myeloblastic leukemia: A case report and literature review

**DOI:** 10.1002/ccr3.7700

**Published:** 2023-07-18

**Authors:** Amirreza Jahanshahi, Sanam Nami, Abbas Ali Hosein Pour Feizi, Samin Alihosseini, Mehran Jaberinezhad, Mirsaeed Abdollahi, Faezeh Rahimi, Masih Falahatian

**Affiliations:** ^1^ Medical Radiation Sciences Research Group Tabriz University of Medical Sciences Tabriz Iran; ^2^ Department of Radiology Tabriz University of Medical Sciences Tabriz Iran; ^3^ Department of Parasitology and Mycology School of Medicine Tabriz University of Medical Sciences Tabriz Iran; ^4^ Hematology and Oncology Research Center Tabriz University of Medical Sciences Tabriz Iran; ^5^ Student Research Committee Tabriz University of Medical Sciences Tabriz Iran; ^6^ Cardiovascular Research Center Tabriz University of Medical Sciences Tabriz Iran; ^7^ Department of Radiology Zanjan University of Medical Sciences Zanjan Iran

**Keywords:** acute myeloid leukemia, Candida albicans, case report, hepatosplenic candidiasis, magnetic resonance imaging, muscular abscess

## Abstract

**Key Clinical Message:**

Muscular and subcutaneous candidiasis is a rare entity in immunocompromised patients, but it should be kept in mind when we see multiple cystic soft tissue masses in addition to target‐shaped hepatosplenic lesions in neutropenic patients. US and MRI are useful imaging modalities for the diagnosis and follow‐up of these patients.

**Abstract:**

Soft tissue candidiasis is an opportunistic infection in immunocompromised patients and must always be diagnosed and treated as soon as possible. In this case report, the patient is a 14‐year‐old boy with acute myeloid leukemia M3‐type who presented with numerous soft tissue and hepatosplenic candidal abscesses.

## Graphical abstract

Soft tissue candidiasis is a type of infection that occurs in individuals with compromised immune systems, and it is crucial to promptly diagnose and treat this condition. We report a 14‐year‐old boy with acute myeloid leukemia M3‐type who presented multiple candidal abscesses in the soft tissues and hepatosplenic regions.

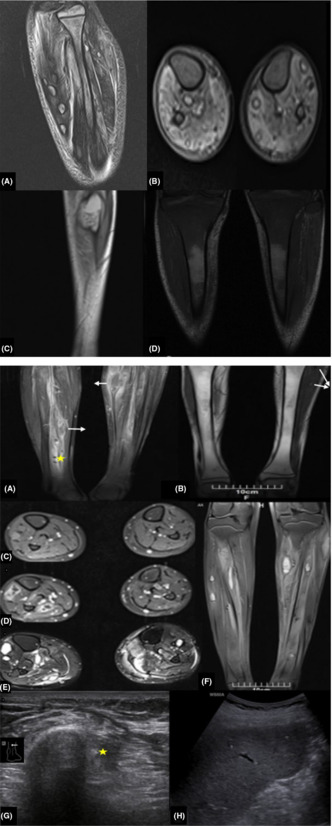

## 1 BACKGROUND

Candida infections are rare in immunocompetent patients. However, it has considerable importance and prevalence in immunocompromised patients, such as transplant and cancer patients.^1^ Patients with cancer are at higher risk of candidiasis, mainly due to chemotherapy‐induced suppression of innate and adaptive immune cells and disruption of epithelial barriers.^2^

The manifestations are usually categorized into mucocutaneous or invasive forms. Typical presentations of the mucocutaneous form can be seen as paronychia, intertrigo, thrush, vulvovaginitis, and esophageal candidiasis. Candidemia is the most easily recognized manifestation of invasive candidiasis, but it can involve virtually any anatomic site and cause widespread visceral dissemination.^3^ However, subcutaneous and intramuscular candidal abscess formation is rare, even in immunocompromised patients, and can be seen as case reports in the literature.^4^ According to our knowledge, this is a rare case of concurrent hepatosplenic candidiasis and Candida albicans muscular abscess in a person with acute myeloid leukemia (AML) type M3.

## 2 CASE PRESENTATION

The patient is a 14‐year‐old boy who has presented with signs of epistaxis and fatigue since 3 months ago. Laboratory data showed severe pancytopenia. Considering the risk of spontaneous hemorrhage, he was immediately transferred to the tertiary center for comprehensive care. Subsequent investigations through bone marrow biopsy and flow cytometry were consistent with the diagnosis of AML M3 type. The patient received appropriate therapy, first with daunorubicin and ATRA and later with Arsenic trioxide. He then presented to the hospital 3 months later with numerous bulging subcutaneous masses on his buttocks, thighs, calves, and plantar surface of his feet. Similar lesions, albeit fewer, were observed in the back and upper extremities.

**FIGURE 1:**
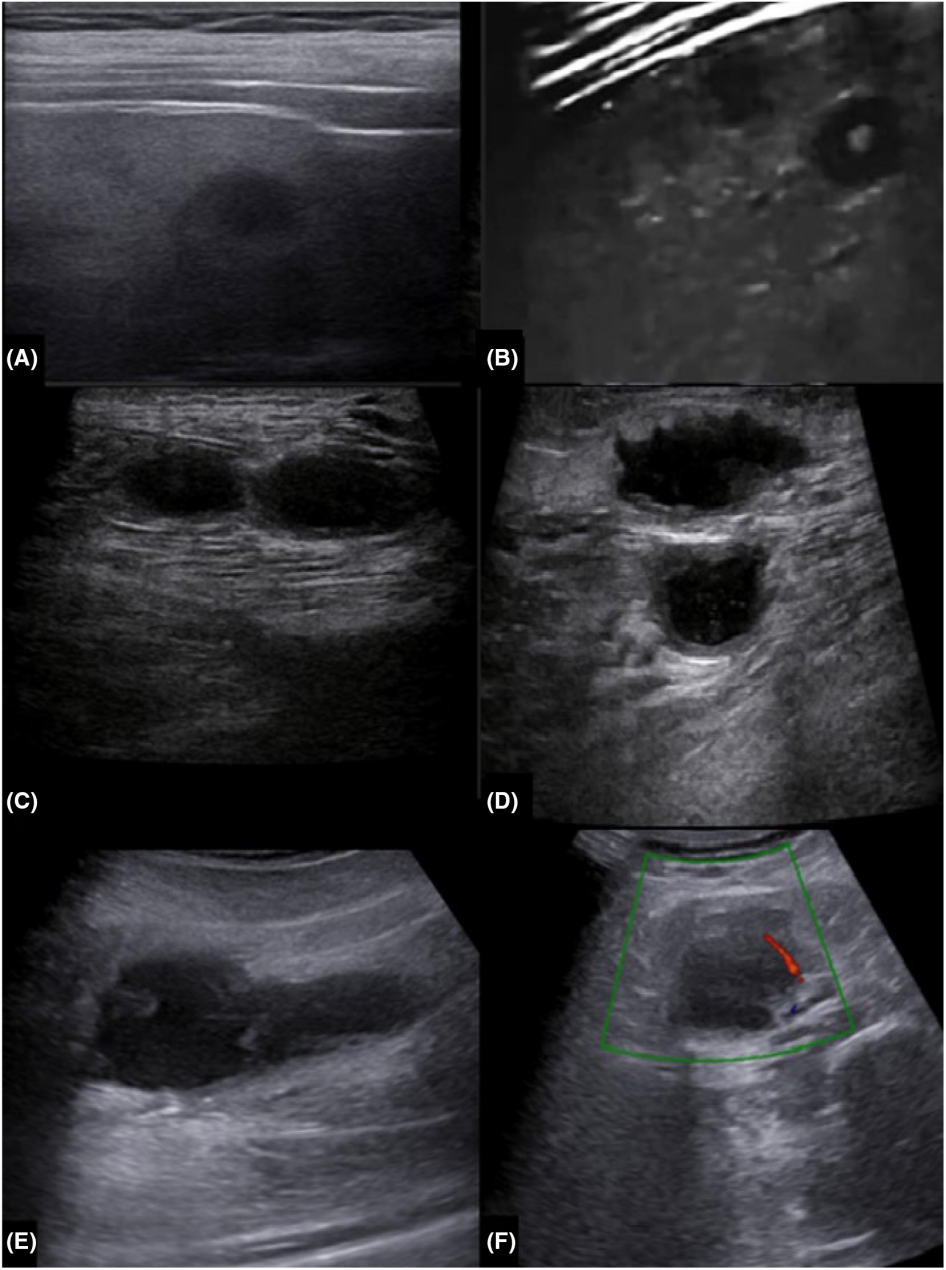
Multiple visceral, intramuscular, and subcutaneous abscesses. Hypoechoic lesion without vascularity in the spleen (A). Hypoechoic and target‐shaped lesions in the periphery of hepatic parenchyma (B) Multiple thick‐walled cystic lesions containing some internal echogenic foci are seen in the subcutaneous area and within the medial muscular compartment of the right leg (C, D). Another intramuscular abscess with peripheral vascularity (E, F).

**FIGURE 2:**
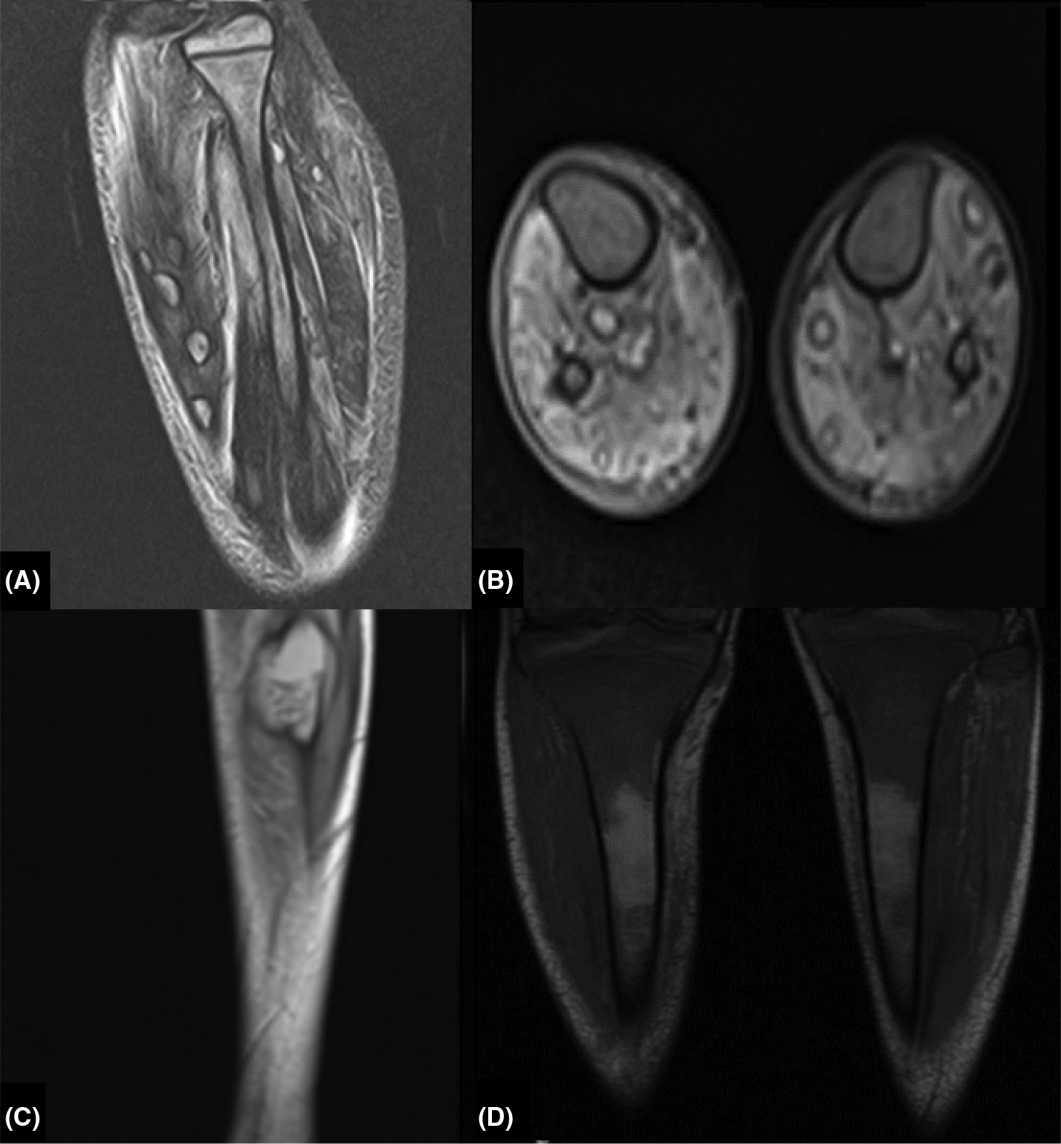
Multiple intramuscular candidal abscesses and leukemic bone marrow infiltration. Coronal T2, fat‐sat and axial T1 fat‐sat images of legs show multiple bilateral hypersignal lesions with hyposignal rim within different muscular compartments (A, B). A similar hypersignal lesion on T2 weighted sequence is seen in the lateral muscular compartment of the left leg, containing linear iso‐ to hyposignal foci (C). Almost diffuse hyposignal intensity is seen in the bone marrow of the bilateral tibia due to leukemic infiltration, proven by bone marrow biopsy (D).

**FIGURE 3:**
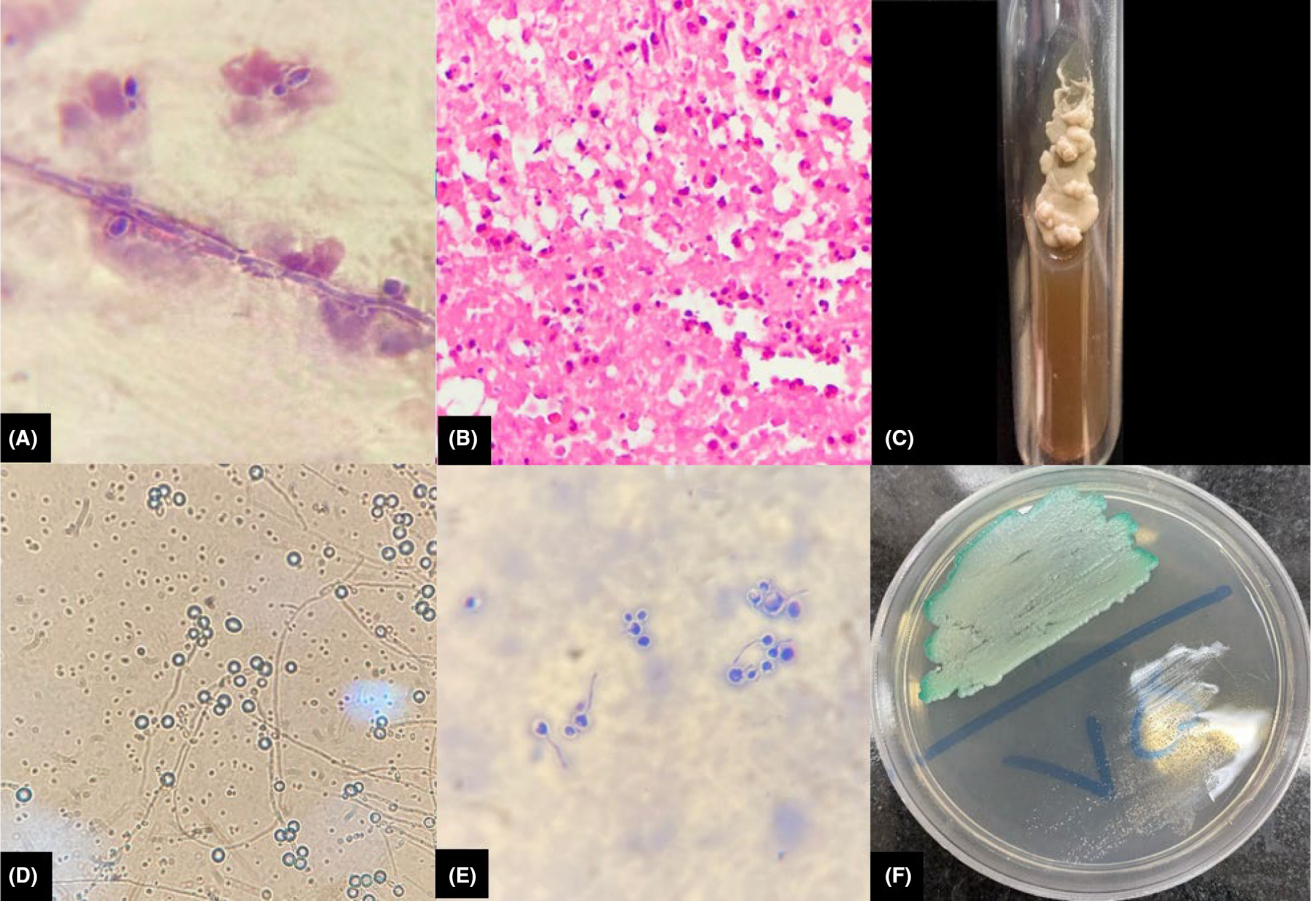
Culture and cytopathological results. Under 100× magnification, gram staining (A) of the subcutaneous abscess's smear revealed yeast cells, pseudohyphae, and budding yeast cells. We did not detect any bacteria or pathogens. Hematoxylin and eosin staining was not conclusive because of severe background inflammation (neutrophil infiltration) and necrosis (B). Samples were inoculated into sabouraud dextrose agar (SDA) with 50 mg chlorampenicol/L (Merek, Germany) and incubated at 30°C for 10 days before being examined directly with 10% potassium hydroxide (KOH). Creamy to white colonies grew on SDA (C), indicating candida species. Micromorphological characteristics, including chlamydospore production (D) on cornmeal agar (High media, India) plus 1% tween 80 (Merek, Germany) and germ tube formation (E) on human serum were suggestive of candida albicans. Sub‐cultures of isolates were then incubated at 35°C for 48 h while being subjected to a chromogenic assay on CHROMagar Candida (F) (CHROMagar, Candida, France) which revealed characteristic light green colonies of candida albicans.

Ultrasonography was done, and multiple thick‐walled cystic lesions containing some internal echogenic material were seen in the subcutaneous tissue and within muscular compartments of the lower extremities with peripheral vascularity on Doppler ultrasound, suggesting of abscess formation. Multiple target‐shaped and hypoechoic lesions were also observed in the liver and spleen (Figure 1). Magnetic resonance imaging (MRI) of the lower extremities was performed and showed numerous iso‐ to hypersignal on T1 and hypersignal on T2‐weighted oval lesions with hyposignal rim within different muscles of the lower extremities and also in subcutaneous tissue. The almost diffuse hyposignal intensity of the bone marrow of the bilateral tibia and fibula on the T1‐weighted sequence was also seen due to leukemic infiltration (Figure 2). Subsequent needle aspiration of muscular lesions under ultrasound guidance was performed, and cytopathology and culture reports were consistent with abscess formation due to *Candida Albicans* (Figure 3). Brain MRI also showed subdural hematoma in the right frontoparietal convexity (due to low platelet level). A chest X‐ray did not show any abnormalities.

**FIGURE 4:**
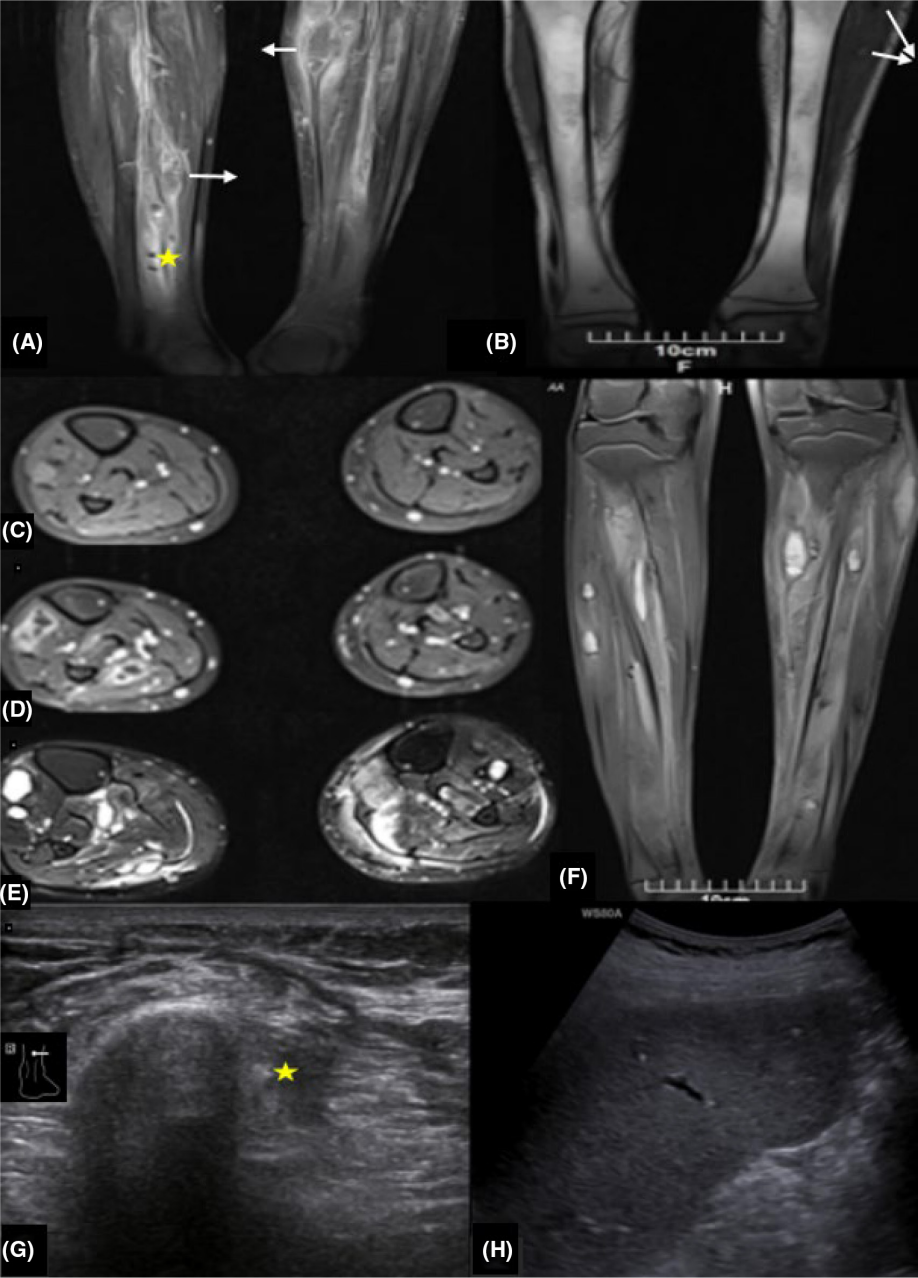
Post‐treatment MRI and ultrasound. After the antifungal treatment, some of the soft tissue abscesses coalesced with each other, some of the lesions disappeared, and some of them calcified. Coronal T1 fat‐sat sequence with contrast shows ring‐enhancing oval‐shaped lesions in the medial muscular compartment of the legs (white arrows). Surrounding edematous tissues also show enhancement. Some foci were signal void on all pulsed sequences and did not show any enhancement (yellow star) (A). Coronal T1 weighted sequence without contrast shows complete resolution of bone marrow infiltration. Two small, oval‐shaped, iso‐ to hypersignal lesions are seen at the lateral muscular compartment of the left leg (white arrows) (B). Axial T1 fat sat without contrast (C), axial T1 fat sat with contrast (D), and axial T2 fat sat images (E) show multiple iso to hypersignal T1 weighted, hypersignal T2‐weighted, oval‐shaped lesions in different muscles of legs, accompanied with ring enhancement. Coronal PD fat sat sequence (F) shows similar hypersignal lesions. Signal void foci on MRI (yellow star in A) were echogenic with faint acoustic shadow in the ultrasound exam (G), indicating calcification (a sign of response to treatment). Hypoechoic splenic lesions (Figure 1A) were calcified after the treatment (H).

So, he was given antifungal therapy with intravenous Amphotericin‐B for 2 weeks, and then, step‐down therapy with oral fluconazole was started. After 3 months of antifungal treatment with oral fluconazole along with chemotherapy, the patient was re‐evaluated clinically and by imaging modalities including ultrasonography and MRI. Compared with pre‐treatment physical examination, he felt generally well, and bulging subcutaneous lesions in the back, upper extremities, and thighs disappeared or shranka. Although MRI showed almost complete resolution of bone marrow leukemic infiltration, many leg abscesses persisted without change, and some of the lesions coalesced together. Fortunately, some leg abscesses changed to non‐enhancing signal void small foci in post‐treatment MRI due to calcification, as confirmed on the targeted ultrasound. Also, the complete resolution of hepatic lesions and calcification of splenic lesions were seen in ultrasonography (Figure 4).

Due to the persistence of abscesses on the buttocks and plantar surface of the feet, which impaired the patient's walking and sitting abilities, a surgical incision was also performed. Post‐surgical pathology was reported as granulation tissue with focal abscess formation and complete resolution of candida infection.

## 3 DISCUSSION

Among AML subtypes, M3 is usually considered highly curable^5^; however, treatment introduces new complications, mainly because of the induction of immunosuppression. Opportunistic infections, particularly fungal infections such as candidiasis, are one of these complications. It is well known that an increase in the fungal load, a compromised mucosal surface, and a lowered host immune response are all necessary for the disease to manifest. Other risk factors can also contribute to this process, namely indwelling catheters and recent surgical and percutaneous interventions.^3^ Candida from intestinal microbial flora typically spreads through blood circulation and causes macro nodular skin lesions. Organisms may spread to other organs, especially the liver, spleen, and kidneys.^6^

Ultrasonography is useful for detecting and monitoring candidiasis lesions; however, candidal lesions may be undetectable in imaging before neutrophil count recovery, especially in chronic disseminated candidiasis (CDC). Manifestations of imaging depend on the stage of the disease, but the most frequent ultrasound pattern in the liver and spleen is several small hypoechoic lesions. Four dominant patterns of hepatosplenic involvement have been described. The earliest pattern comprises a peripheral hypoechoic zone that correlates with fibrosis, with a second hyperechoic zone consisting of inflammatory cells. The central hypoechoic nidus relates to necrotic fungal elements. This pattern is called the “wheel within a wheel.” The second pattern is called the “bull's eye” or target pattern, with a peripheral hypoechoic halo encircling a central echogenic core. The third and most common pattern is seen as multiple hypoechoic lesions that can be seen in conjunction with the other three patterns. The fourth pattern, manifesting as echogenic foci, is usually seen at the late stages of the disease and correlates microscopically with central fibrosis, calcifications, or both.^7^

MRI seems superior to computed tomography (CT) scan and ultrasonography in identifying hepatosplenic and musculoskeletal lesions associated with candidiasis. In a patient with acute hepatosplenic or soft tissue candidiasis, lesions on MRI are round, measured <1 cm in diameter, markedly hyperintense on T2‐weighted images, and show restriction on diffusion‐weighted imaging (DWI). At the chronic stage, especially with antifungal treatment, a hyposignal rim surrounding the primary lesions and a non‐enhancing center on contrast images are seen, consistent with the necrotic core seen on histologic examination. When the lesions are calcified, they appear hyperdense in CT scan and have low signal in MRI.^8^, ^9^, ^10^

There aren't many studies that provide imaging results for subcutaneous and intramuscular candidal abscesses. We discovered a few case reports in the literature that contained imaging data such as CT or MRI (Table 1). When dealing with an AML patient who has various cutaneous, muscular, and hepatosplenic lesions, we must evaluate a variety of differential diagnoses, including chloroma, multifocal bacterial abscess, tuberculoma, cysticercosis, and hydatidosis, in addition to systemic candidiasis.

**TABLE 1:** Cases of subcutaneous and muscular abscesses caused by *Candida albicans* published in the literature.

Author (Year)	Sex/age	Risk factors for Candida infection	Underlying medical condition	Localization	Imaging findings (Ultrasound, CT, MRI)	Treatment	Surgery
Nelp^27^ (1963)	17/F	Poorly controlled diabetes mellitus, Daily injection of insulin	Diabetes mellitus	Thighs	**_**	Nitrofurazone gauze in abscesses cavities	+
Mochizuki et al.^4^ (1988)	59/M	2‐month hospitalization (bedridden)	Subarachnoid hemorrhage, intracranial surgery (4 times)	Left Knee (3 × 11 cm subcutaneous abscess)	**_**	Miconazole, Amphotericin‐B	−
Manfredi et al.^28^ (1997)	36/M	Progressive HIV‐related immunocompromise, IV‐drug abuser, peripheral sensory neuropathy, advanced liver cirrhosis, chronic HBV infection (treated with zidovudine (500 mg daily) for 6 months)	AIDS	Right upper thoracic wall (5 cm)	Chest X‐ray and an ultrasonographic examination confirmed an isolated subcutaneous abscess	Oral itraconazole	_
Tuon et al.^29^ (2006)	32/M	Tuberculosis	Gastrointestinal surgery, short bowel syndrome	Skin abscess	−	Fluconazole	+
Florescu et al.^30^ (2010)	50/M	Immunosuppression (Mycophenolate mofetil 1.5 g twice a day, prednisone 5 mg/d and a day, prednisone 5 mg/d	Cardiac transplant recipient, on trimethoprim/sulfamethoxazole treatment for cerebral nocardiosis	Subcutaneous abscesses of legs	MRI: cellulites and a medial fluid collection (4* 1.5 cm) CT: large (5*3*7 cm) multiloculated fluid collection in the posterior leg	Moxifloxacin, fluconazole; trimethoprim/sulfamethoxazole	_
Buchanan et al.^31^ (2013)	55/M	Central catheter placement	Gunshot wounds	Neck	CT: multiseptated abscess of the left lower neck and left supraclavicular region	Anidulafungin	+
Kakeya et al.^32^ (2014)	50/M	Immunosuppression (40 mg/day of hydrocortisone, 0.5 mg/day of dexamethasone)	Right adrenalectomy, daily corticosteroid replacement, ushing's syndrome, pulmonary cryptococcosis	Subcutaneous abscesses of legs	MRI: rounded fluid collection signal in the soft tissue of the legs	Fluconazole	+
Musa et al.^33^ (2014)	67/M	Diabetes mellitus	−	Retropharyngeal region	Lateral neck x‐ray: widening of soft tissue of prevertebral area with area of lucency indicating air content CT: ill‐defined thin rim‐enhancing hypodense collection	Oral fluconazole	+
Peker et al.^34^ (2015)	68/M	Diabetes mellitus	Buccal‐space infection	Left cheek	Panoramic radiograph: extensive bone loss between the teeth related with buccal abscess MRI: fluid accumulation compatible with an abscess located in the area of masticatory muscle structures reaching the left infraorbital region	Amphotericin‐B and oral fluconazole	_
Messina et al.^35^ (2015)	42/M	Immunosuppression	HIV‐seropositive	Right chest wall	Ultrasound: heterogeneous mass with irregular contours with echogenic stippling and hypo echoic area in intimate contact with the muscular plane CT: liquid collection in relation to the anterior chest wall with air bubbles	Fluconazole	+
Kothari et al.^36^ (2016)	42/M	Chemotherapy (darubicin, cytarabine and cytarabine), neutropenic	Acute myeloid leukemia	Arms, chest, back, and legs, spleen	CT: splenomegaly and multiple hypodense nodules FDG PET: splenomegaly with diffuse uptake in the spleen	Micafungin, voriconazole	_
Amita et al.^37^ (2017)	60/M	Diabetes mellitus	Tuberculosis	Right forearm	−	Intravenous antifungal drug	_
Yi et al.^38^ (2019)	60/M	Myelosuppressive chemotherapy	Acute granulomonocytic leukemia	Subcutaneous abscess of calves, splenic abscess	CT: multiple hypodense lesions in the spleen MRI: multiple subcutaneous abscesses in the calves	Amphotericin‐B flucytosine	_
Sung et al.^39^ (2021)	57/F	Type 2 diabetes mellitus	Blunt eyelid trauma with self‐administered acupuncture	Facial candidal abscess	CT: soft tissue swelling in the left periorbital area	Fluconazole	+
Yoshihara et al.^40^ (2022)	42/F	Hashimoto's disease	Laparoscopic cholecystectomy 9 months prior to admission	Lower left rib in the anterior chest wall	Contrast‐enhanced CT: the fluid density area around the lower left side of rib (6.5 cm in diameter)	Micafungin, fluconazole	+
Current study (2023)	14/M	Acute myeloid leukemia M3 type (Treated with daunorubicin, ATRA and later with Arsenic trioxide)	Acute myeloid leukemia M3 type	Arms, buttocks, thighs, calves, and plantar surface of feet	Ultrasonography: multiple thick‐walled cystic lesions containing some internal echogenic material, Multiple target shaped and some hypoechoic lesions were also observed in liver and spleen MRI: numerous iso to hyper signal on T1 and hyper signal on T2 weighted oval lesions with hyposignal rim within different muscles	Amphotericin‐B and fluconazole	+

Chloromas or myeloid sarcomas comprise immature myeloid cells, most often leukemic blasts.^11^ It is characterized by an extramedullary tumoral lesion which can readily be diagnosed by ultrasonography, CT scan, and biopsy.^12^ The numbers of these lesions are usually lower than candidal lesions. In MRI, they present as iso‐signal or hyposignal on T1 and mildly hypersignal on T2‐weighted images. They have vascularity in Doppler ultrasound and show enhancement after contrast injection in CT scan and MRI.^12^ Bone and periosteum are the most common sites of involvement, but any tissue can be affected, such as skin, orbit, paranasal sinuses, and the central nervous system.^13^, ^14^ Moreover, chloroma is more prevalent in AML M2, M4, and M5 subtypes, not M3.^15^

Multifocal bacterial abscesses can occur in immunocompromised patients. Septic emboli can be primarily found in the lungs, especially in AML patients with port catheter.^16^ However, culture and gram staining of blood and aspirated abscess fluid returned negative for our patient; his chest X‐ray was also normal. Moreover, bacterial abscesses do not show the typical “bull's eye” ultrasound pattern.

Extra‐pulmonary tuberculosis should always be considered as a differential diagnosis of multiple subcutaneous and hepatosplenic masses in an immunocompromised patient, even though it's a rare finding.^17^ These abscesses are often observed in the chest wall and spine. The limb is a very uncommon location of involvement.^18^ They are usually secondary to ruptured necrotic lymph nodes, tuberculous osteomyelitis, or arthritis.^19^ Culture and acid‐fast staining of blood and abscess fluid were also negative.

Cysticercosis is a kind of endemic parasitic disease that is rare in our country. The most commonly affected tissues are the central nervous system and skeletal muscles. In ultrasonography, the scolex is seen inside the lesion, which may be calcified. When the lesions are cystic, they have similar characteristics to fluid on both CT and MRI, but when these lesions calcify, they appear as hyperdense foci parallel to muscle fibers on CT, giving a characteristic appearance called “rice‐grain” calcification.^20^ This disease is not related to the host's immunity state.^21^

Soft tissue and skeletal muscle hydatid cysts are very rare and usually secondary. They can occur in the lower extremities, trunk, neck, or legs. The pectoralis major, sartorius, quadriceps, and gluteus muscles can be involved. It usually appears as a focal multi‐vesicular cystic lesion in the muscle(s) that can invade the adjacent bone.^22^ They have a characteristic appearance on ultrasound, CT scan, and T2 weighted sequence of MRI as a cystic lesion with serpentine undulant membranes called “water lily” sign or “serpentine” sign in the liver, spleen, and other regions.^23^

Soft tissue mycetomas due to maduramycosis or other fungal infections usually occur in the foot and endemic areas. Mycetoma is a kind of chronic soft tissue inflammation caused by fungi or actinomycetes. They appear as multiple, small, round T2 hyperintense lesions with central hypointense foci in MRI. Central hypointense foci are mycetoma grains in pathology called the “dot in a circle” sign which is specific for this entity.^24^, ^25^ One of our patient's lesions had a similar appearance in MRI (Figure 2C). Although the “dot in a circle” sign is characteristic for mycetoma, the accumulation of candida hyphae can cause hypointense signal areas in both T2 and T1 weighted images in MRI.^26^

## 4 CONCLUSION

In conclusion, we presented an AML M3 patient with a multifocal muscular and hepatosplenic abscess caused by Candida albicans, shown in ultrasound and MRI and proved by pathology. He was treated successfully with medical and surgical methods. Also, we reviewed the literature about the imaging manifestations of a few similar cases and finally discussed the imaging features of musculoskeletal candidiasis and its differential diagnosis.

## AUTHOR CONTRIBUTIONS

**Amirreza Jahanshahi:** Investigation; supervision; validation; visualization. **Sanam Nami:** Validation; visualization. **Abbas Ali Hosein Pour Feizi:** Investigation; validation. **Samin Alihosseini:** Visualization; writing – original draft; writing – review and editing. **Mehran Jaberinezhad:** Writing – original draft; writing – review and editing. **Mirsaeed Abdollahi:** Writing – original draft; writing – review and editing. **Faezeh Rahimi:** Writing – original draft. **Masih Falahatian:** Conceptualization; investigation; project administration; visualization; writing – review and editing.

## FUNDING INFORMATION

None.

## CONFLICT OF INTEREST STATEMENT

No author states to have any conflicts of interest.

## PATIENT CONSENT STATEMENT

Written informed consent was obtained from the patient for publication of this case report and accompanying images.

## Data Availability

The datasets supporting the conclusions of this article is(are) included within the article and its additional files.

## References

[1] Kao AS , Brandt ME , Pruitt WR , et al. The epidemiology of candidemia in two United States cities: results of a population‐based active surveillance. Clin Infect Dis. 1999;29(5):1164‐1170. doi:10.1086/313450 10524958

[2] Teoh F , Pavelka N . How chemotherapy increases the risk of systemic candidiasis in cancer patients: current paradigm and future directions. Pathogens. 2016;5(1):6. doi:10.3390/pathogens5010006 26784236 PMC4810127

[3] McCarty TP , White CM , Pappas PG . Candidemia and invasive candidiasis. Infect Dis Clin North Am. 2021;35(2):389‐413. doi:10.1016/j.idc.2021.03.007 34016283

[4] Mochizuki T , Urabe Y , Hirota Y , Watanabe S , Shiino A . A case of Candida albicans skin abscess associated with intravenous catheterization. Dermatology. 1988;177(2):115‐119. 10.1159/000248526 3169333

[5] Stahl M , Tallman MS . Acute promyelocytic leukemia (APL): remaining challenges towards a cure for all. Leuk Lymphoma. 2019;60(13):3107‐3115. doi:10.1080/10428194.2019.1613540 31842650 PMC7479633

[6] Strickland AB , Shi M . Mechanisms of fungal dissemination. Cell Mol Life Sci. 2021;78(7):3219‐3238. doi:10.1007/s00018-020-03736-z 33449153 PMC8044058

[7] Görg C , Weide R , Schwerk W , Köppler H , Havemann K . Ultrasound evaluation of hepatic and splenic microabscesses in the immunocompromised patient: sonographic patterns, differential diagnosis, and follow‐up. J Clin Ultrasound. 1994;22(9):525‐529. 7806659 10.1002/jcu.1870220902

[8] Semelka RC , Kelekis N , Sallah S , Worawattanakul S , Ascher S . Hepatosplenic fungal disease: diagnostic accuracy and spectrum of appearances on MR imaging. AJR Am J Roentgenol. 1997;169(5):1311‐1316. 9353448 10.2214/ajr.169.5.9353448

[9] Anttila VJ , Lamminen AE , Bondestam S , et al. Magnetic resonance imaging is superior to computed tomography and ultrasonography in imaging infectious liver foci in acute leukaemia. Eur J Haematol. 1996;56(1–2):82‐87. 8600000 10.1111/j.1600-0609.1996.tb00300.x

[10] Kelekis NL , Semelka RC , Jeon H‐J , Sallah AS , Shea TC , Woosley JT . Dark ring sign: finding in patients with fungal liver lesions and transfusional hemosiderosis undergoing treatment with antifungal antibiotics. Magn Reson Imaging. 1996;14(6):615‐618. 8897364 10.1016/0730-725x(96)00090-2

[11] Shallis RM , Gale RP , Lazarus HM , et al. Myeloid sarcoma, chloroma, or extramedullary acute myeloid leukemia tumor: a tale of misnomers, controversy and the unresolved. Blood Rev. 2021;47:100773. doi:10.1016/j.blre.2020.100773 33213985

[12] Singh A , Kumar P , Chandrashekhara SH , Kumar A . Unravelling chloroma: review of imaging findings. Br J Radiol. 2017;90(1075):20160710. doi:10.1259/bjr.20160710 28445074 PMC5594979

[13] Yilmaz AF , Saydam G , Sahin F , Baran Y . Granulocytic sarcoma: a systematic review. Am J Blood Res. 2013;3(4):265‐270. 24396704 PMC3875275

[14] Shinagare AB , Krajewski KM , Hornick JL , et al. MRI for evaluation of myeloid sarcoma in adults: a single‐institution 10‐year experience. Am J Roentgenol. 2012;199(6):1193‐1198. 23169708 10.2214/AJR.12.9057

[15] McCarty SM , Kuo DJ . Persistent sacral chloroma in refractory acute myelogenous leukaemia. BMJ Case Rep. 2017;2017:bcr‐2017‐219936. 10.1136/bcr-2017-219936 PMC5534900 28687689

[16] Eckmann C . The importance of source control in the management of severe skin and soft tissue infections. Curr Opin Infect Dis. 2016;29(2):139‐144. 26779777 10.1097/QCO.0000000000000240

[17] Sezgin B , Atilganoglu U , Yigit O , Ergün SS , Cambaz N , Demirkesen C . Concomitant cutaneous metastatic tuberculous abscesses and multifocal skeletal tuberculosis. Indian J Dermatol. 2008;53(3):149. 19882018 10.4103/0019-5154.43208 PMC2763739

[18] Hussain S . Chest wall tuberculous ulcer: a rare complication of pulmonary tuberculosis. Indian J Tuberc. 2016;63(4):265‐267. 27998501 10.1016/j.ijtb.2015.05.003

[19] De Backer A , Mortelé K , Vanhoenacker F , Parizel P . Imaging of extraspinal musculoskeletal tuberculosis. Eur J Radiol. 2006;57(1):119‐130. 16139465 10.1016/j.ejrad.2005.07.005

[20] Pankaj N , Vijayanadh O . Rice‐grain calcifications of cysticercosis. Abdom Radiol. 2021;46(3):1276‐1277. 10.1007/s00261-020-02777-z 32939633

[21] Venkat B , Aggarwal N , Makhaik S , Sood R . A comprehensive review of imaging findings in human cysticercosis. Jpn J Radiol. 2016;34(4):241‐257. 26903229 10.1007/s11604-016-0528-4

[22] Gougoulias N , Varitimidis S , Bargiotas K , Dovas T , Karydakis G , Dailiana Z . Skeletal muscle hydatid cysts presenting as soft tissue masses. Hippokratia. 2010;14(2):126‐130. 20596270 PMC2895288

[23] El‐Tahir M , Omojola M , Malatani T , Al‐Saigh A , Ogunbiyi O . Hydatid disease of the liver: evaluation of ultrasound and computed tomography. Br J Radiol. 1992;65(773):390‐392. 1611417 10.1259/0007-1285-65-773-390

[24] Basirat A , Boothe E , Mazal AT , Mansoori B , Chalian M . Soft tissue mycetoma: “Dot‐in‐circle” sign on magnetic resonance imaging. Radiol Case Rep. 2020;15(5):467‐473. 32123555 10.1016/j.radcr.2020.01.024 PMC7036743

[25] Jain V , Makwana GE , Bahri N , Mathur MK . The “dot in circle” sign on MRI in maduramycosis: a characteristic finding. J Clin Imaging Sci. 2012;2:66. 23230548 10.4103/2156-7514.103056 PMC3515922

[26] Ruiz–Cabello J , Carrero–González B , Avilés P , et al. Magnetic resonance imaging in the evaluation of inflammatory lesions in muscular and soft tissues: an experimental infection model induced by Candida albicans. Magn Reson Imaging. 1999;17(9):1327‐1334. 10576718 10.1016/s0730-725x(99)00061-2

[27] Nelp WB . Multiple Candida abscesses resulting from insulin injections. N Engl J Med. 1963;268(12):664‐665. doi:10.1056/nejm196303212681208 13938015

[28] Manfredi R , Mazzoni A , Nanetti A , Mastroianni A , Coronado OV , Chiodo F . Isolated subcutaneous Candidal abscess and HIV disease. Br J Dermatol. 1997;136(4):647‐649. doi:10.1111/j.1365-2133.1997.tb02177.x 9155990

[29] Tuon FF , Nicodemo AC . Candida albicans skin abscess. Rev Inst Med Trop Sao Paulo. 2006;48:301‐302. 17086322 10.1590/s0036-46652006000500012

[30] Florescu DF , Brostrom SE , Dumitru I , Kalil AC . Candida albicans skin abscess in a heart transplant recipient: case report and review of the literature. Infect Dis Clin Pract. 2010;18(4):243‐246.

[31] Buchanan PJ , Mast BA , Lottenberg L , Kim T , Efron PA , Ang DN . Candida albicans necrotizing soft tissue infection: a case report and literature review of fungal necrotizing soft tissue infections. Ann Plast Surg. 2013;70(6):739‐741. 23123606 10.1097/SAP.0b013e31823fac60

[32] Kakeya H , Izumikawa K , Yamada K , et al. Concurrent subcutaneous candidal abscesses and pulmonary cryptococcosis in a patient with diabetes mellitus and a history of corticosteroid therapy. Intern Med. 2014;53(12):1385‐1390. 24930663 10.2169/internalmedicine.53.1409

[33] Musa Z , Mohamad I , Johan KB , Harun A . Candida albicans retropharyngeal abscess. Pak J Otolaryngol. 2014;30:29‐31.

[34] Peker E , Zor F , Toprak ME , Bariş E . Facial Candidal abscess in a patient with unknown type 2 diabetes mellitus. J Maxillofac Oral Surg. 2015;14(4):995‐998. 26604475 10.1007/s12663-014-0680-2 PMC4648778

[35] Messina F , Negroni R . Subcutaneous abscess as a single manifestation of candidiasis. Med Mycol. 2015;1(1):6.

[36] Kothari A , Shalin SC , Crescencio JCR , Burgess MJ . Skin lesion with splenic microabscesses in a patient with acute myeloid leukemia. Am J Med. 2016;129(12):e325‐e327. 27637597 10.1016/j.amjmed.2016.08.026

[37] Amita K , Govind AM , Pechiat T , Manchaih S , Shankar SV . Candida Albicans infection masquerading as a soft tissue tumour diagnosed by fine needle aspiration cytology. J Clin Diagn Res. 2017;11(7):ED18. 10.7860/JCDR/2017/26062.10227 PMC5583920 28892913

[38] Yi P , Yang X , Yang M , Zhang Y , Li L . Multifocal muscle candidiasis of the legs in a patient with acute myeloid leukemia: a case report. Medicine. 2019;98(8):e14580. 30813175 10.1097/MD.0000000000014580 PMC6408043

[39] Sung JY , Kim JM , Lee JU , Lee YH , Lee SB . Multiple facial candidal abscesses after self‐administered acupuncture in a patient with undiagnosed diabetes mellitus: a case report. BMC Complement Med Ther. 2021;21(1):1‐4. 34112168 10.1186/s12906-021-03343-w PMC8193879

[40] Yoshihara H , Kurihara I , Hori H , Fukuchi T , Sugawara H . Subcutaneous chest abscess caused by Candida albicans infection following laparoscopic cholecystectomy in an immunocompetent patient: a case report. Cureus. 2022;14(4):e24573. doi:10.7759/cureus.24573 35664401 PMC9148386

